# Non-Contrast CT Hemorrhage Markers and Outcomes in Intracerebral Hemorrhage: A Large Single-Center Cohort from Romania

**DOI:** 10.3390/reports8030159

**Published:** 2025-08-28

**Authors:** Cosmin Cindea, Vicentiu Saceleanu, Patrick Canning, Corina Roman-Filip, Romeo Mihaila

**Affiliations:** 1 Faculty of Medicine, Lucian Blaga University of Sibiu, 550024 Sibiu, Romania; vicentiu.saceleanu@gmail.com (V.S.); corina.roman@ulbsibiu.ro (C.R.-F.); romeo.mihaila@ulbsibiu.ro (R.M.); 2 Neurosurgery Departament, County Clinical Emergency Hospital of Sibiu, 550245 Sibiu, Romania; 3 Limerick University Hospital, V94 F858 Limerick, Ireland; patrickcanning9@gmail.com; 4 Neurology Departament, County Clinical Emergency Hospital of Sibiu, 550245 Sibiu, Romania; 5 Hematology Department, County Clinical Emergency Hospital of Sibiu, 550245 Sibiu, Romania

**Keywords:** intracerebral hemorrhage, non-contrast CT, hematoma expansion, radiological signs

## Abstract

Background and Purpose: Spontaneous intracerebral hemorrhage (ICH) is associated with high rates of morbidity and mortality. Early hematoma expansion (HE) is a key driver of poor outcomes, yet readily available non-contrast CT (NCCT) markers remain underused. We assessed four predefined NCCT signs—Blend Sign (BS), Black Hole Sign (BHS), Irregular Shape (IRS), and Satellite Sign (SS)—and a simple composite score (SUM_BBIS, 0–4) for their association with HE and in-hospital mortality. Methods: We retrospectively analyzed 404 consecutive adults with primary spontaneous ICH admitted to a tertiary-care center between January 2017 and December 2023. Patients with secondary causes of hemorrhage or without follow-up NCCT were excluded. Each sign was scored dichotomously by blinded readers and summed to form the SUM_BBIS. HE was defined as a >6 mL or >33% volume increase on repeat NCCT within 24–48 h. Outcomes included HE and in-hospital mortality; secondary analyses explored relationships with baseline hematoma volume, location, intraventricular extension (IVH), and comorbidities. Results: Among 404 patients, Irregular Shape was most frequent (62.1%), followed by Satellite Sign (34.9%), Black Hole Sign (31.1%), and Blend Sign (15.3%). Hematoma expansion occurred in 22.0% (89/404). Expansion was more common when ≥1 sign was present, with the Black Hole Sign showing the strongest association (56.2% vs. 23.8%; *p* < 0.001). In-hospital mortality rose stepwise with higher SUM_BBIS (mean 1.95 in non-survivors vs. 0.93 in survivors; *p* < 0.001). Conclusions: The four predefined NCCT signs, particularly BHS, identify ICH patients at increased risk of HE and in-hospital death. A simple, purely imaging-based composite (SUM_BBIS) captures cumulative radiological complexity and stratifies risk in a stepwise manner. Systematic evaluation of these markers may enhance early triage and inform timely therapeutic decisions, especially in emergency and resource-limited settings.

## 1. Introduction

Spontaneous intracerebral hemorrhage (ICH) remains a serious subtype of stroke, accounting for approximately 10–15% of all strokes worldwide and leading to significant morbidity and mortality [1]. Unlike ischemic stroke, ICH often causes increased intracranial pressure, perihematomal edema, and hematoma expansion, leading to neurological deterioration and high case fatality. Early hematoma expansion (HE), commonly defined as a ≥6 mL or ≥33% volume increase, occurs in ~20–35% of patients, mainly within the first 24 h [2]. This phenomenon is one of the most important predictors of in-hospital mortality and poor functional outcome [3].

Several imaging-based risk factors for HE and poor prognosis have been identified. Among them, the “Spot Sign” on CT angiography (CTA) has been extensively studied as a predictor of hematoma expansion [4]. However, performing CTA in all ICH cases is not always feasible due to clinical, logistical, or resource-related constraints. Therefore, attention has increasingly turned to specific signs on non-contrast CT (NCCT), which is universally performed in acute ICH, to predict progression. Four such NCCT-based signs described in the literature are the Blend Sign, the Black Hole Sign, Irregular Shape, and the Satellite Sign [5].

Each of these signs, when present on initial NCCT, has been associated with higher risk of hematoma growth and worse neurological outcomes. However, there remain questions about their relative frequency and about how they co-occur in a typical unselected ICH population.

We undertook this single-center cohort study to achieve the following:

1. Determine the frequency of the Blend Sign, Black Hole Sign, Irregular Shape, and Satellite Sign in a consecutive population of ICH patients.
2. Evaluate how these signs relate to hematoma characteristics, such as location, volume, and intraventricular extension.
3. Examine their association with ICH progression (i.e., hematoma expansion) and in-hospital mortality.
4. Assess a simple additive score—the sum of the four signs, “SUM_BBIS” (0–4)—to gauge the overall “radiological instability” of the hematoma and its possible value in prognostication.

The novelty of this study lies in evaluating these previously described NCCT signs collectively and integrating them into a composite score (SUM_BBIS), specifically within a Romanian patient cohort, to provide valuable regional and clinical insights.

Existing expansion prediction approaches often incorporate clinical or temporal variables [5]; however, onset time is frequently uncertain in unwitnessed ICH. In contrast, SUM_BBIS comprises imaging only, quantifying NCCT heterogeneity without external inputs and remaining applicable in emergency or resource-limited settings.

Reported prevalence and prognostic strength vary across studies, likely reflecting differences in imaging protocols, sign definitions, and cohort selection [6]. Most analyses emphasize single markers or mix imaging with clinical/temporal variables, limiting generalizability when onset time is unknown. The incremental value of a purely imaging-based composite in an unselected ICH population remains unclear, motivating the present study.

Evaluating these markers in Romania is clinically relevant given regional risk profiles (e.g., hypertension) [7], anticoagulant use patterns (including VKA) [8], imaging access, and treatment timelines that may affect sign distribution and prognostic value.

We hypothesized that higher SUM_BBIS scores would correlate with larger baseline volume and be independently associated with both hematoma expansion and in-hospital mortality.

## 2. Materials and Methods

### 2.1. Study Design and Patient Selection

Between January 2017 and December 2023, we retrospectively screened all spontaneous ICH admissions at a single tertiary medical center (*n* = 602). Exclusions included secondary hemorrhages (vascular malformations, aneurysms, tumors, or hemorrhagic transformation of ischemic stroke), inadequate baseline NCCT, primary intraventricular hemorrhage without a parenchymal component, and no follow-up CT within 24–48 h. The final cohort comprised 404 patients, each with a high-quality baseline NCCT in PACS plus follow-up imaging. Clinical data from electronic health records included demographics (age, sex), hypertension, diabetes, chronic kidney disease, chronic alcohol use, and antithrombotic therapy (vitamin K antagonists, direct oral anticoagulants [DOACs], or antiplatelets). Patients who underwent surgery after their follow-up NCCT were retained in all analyses; those operated on before the follow-up scan were excluded from hematoma expansion analyses to avoid measurement bias, but their baseline data were retained for descriptive purposes.

### 2.2. Imaging Protocol and Definitions of Radiological Signs

All eligible patients underwent a baseline NCCT scan upon presentation, followed by a second NCCT within 24–48 h or sooner if clinical deterioration occurred, to detect hematoma expansion. Four key radiological signs were evaluated on the initial NCCT.

**Figure 1:**
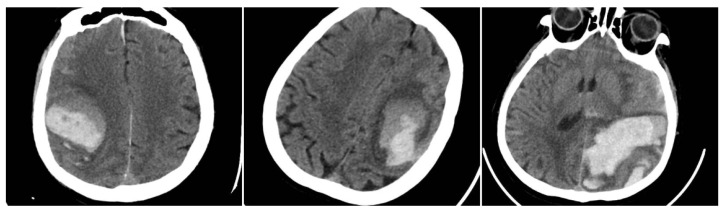
(**Left**) Hematoma showing Black Hole and Blend Signs; (**middle** and **right**) other examples of Blend Signs.

The Blend Sign (BS) was recorded when a well-defined hypoattenuating region directly abutted a hyperattenuating area within the same hematoma, differing by ≥18 HU, without the hypodense portion being fully encapsulated by the hyperdense region (BS = 1 if present, otherwise 0) (Figure 1) [9].

**Figure 2:**
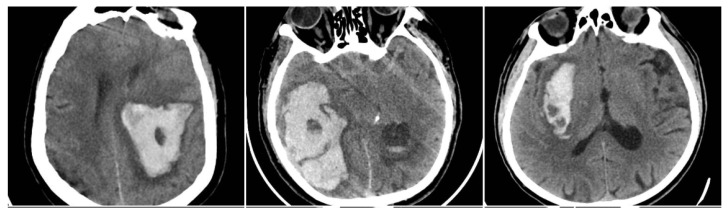
Black Hole Sign (BHS)—three examples from our cohort.

The Black Hole Sign (BHS) refers to a discrete, well-defined hypoattenuating region fully enclosed within a hyperdense clot with ≥28 HU difference and no continuity with normal parenchyma (BHS = 1 if present, otherwise 0) (Figure 2) [10].

**Figure 3:**
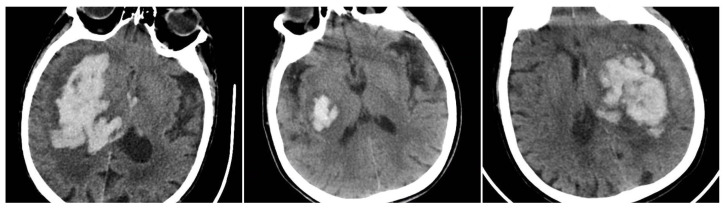
Irregular Shape sign—three examples from our cohort.

Irregular Shape (IRS) has a clearly lobulated or spiculated hematoma margin, interpreted as multifocal bleeding sources (IRS = 1 if irregular, otherwise 0) (Figure 3) [11].

**Figure 4:**
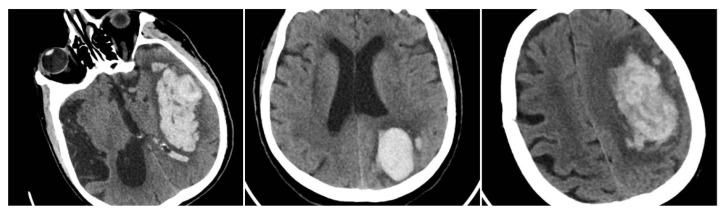
Satellite Sign (SS)—three examples from our cohort.

The Satellite Sign (SS) was identified when a small (≤10 mm) hyperdense hemorrhagic focus lay within 20 mm of, but separate from, the main clot on at least one CT slice (SS = 1 if present, otherwise 0) (Figure 4) [12].

A composite score, SUM_BBIS = BS + BHS + IRS + SS (range 0–4), was calculated for each patient. Two blinded raters assessed all signs independently; disagreements were resolved through consensus, with substantial agreement (κ > 0.70).

### 2.3. Hematoma Volume, Location, and Progression

All 404 patients underwent repeat NCCT to assess size change. Hematoma progression was defined as >33% or >6 mL increase from baseline (coded YES/NO). Volume was independently estimated by two neuroradiologists using the ABC/2 ellipsoid method, where A = largest axial diameter, B = perpendicular diameter on the same slice, C = slice thickness × number of slices with blood; volume ≈ (A × B × C)/2 [13,14]. Location was assigned based on the deepest structure involved (capsulo-lenticular, thalamic, lobar, cerebellar, or brainstem). Intraventricular extension (IVH) was recorded when blood was visible in the ventricles on baseline or follow-up CT. Management was classified as conservative or surgical [15]; surgical treatment comprised craniotomy or minimally invasive evacuation and termed early if performed ≤48 h from baseline CT and delayed if >48 h.

### 2.4. Outcome Measures

Primary outcomes included the overall frequency of each sign, the distribution of SUM_BBIS scores, and the association of these signs with hematoma progression. We also examined in-hospital mortality and explored differences in sign prevalence between key clinical subgroups, such as surgical vs. conservative management and anticoagulant vs. non-anticoagulant therapy.

### 2.5. Statistical Analysis

Analyses were performed in SPSS v26.0 (IBM, Armonk, NY, USA). Univariate comparisons were conducted in the full cohort and within conservative and surgical strata [16]. Two multivariable logistic regression models evaluated (i) hematoma expansion and (ii) in-hospital mortality, including age, baseline hematoma volume, intraventricular hemorrhage (IVH), anticoagulation status, each NCCT sign, management strategy, and the management×NCCT-sign interaction. Model performance was summarized with Nagelkerke R^2^, ROC AUC, and Hosmer–Lemeshow χ^2^ (*p* > 0.05 acceptable) with residual diagnostics; multicollinearity was assessed through VIF (<2 for all predictors).

Missing data were profiled. Variables with <5% missingness were analyzed using complete-case analysis; those with ≥5% were imputed (median for continuous, mode for categorical). Overall missingness in the final models was <5%. Prespecified sensitivity analyses included (i) excluding follow-up CTs >48 h; (ii) excluding patients operated on before follow-up imaging; and (iii) re-fitting models excluding anticoagulated patients. Effect sizes and significance were stable. We also tested sex×marker and sex×SUM_BBIS interactions and performed sex-stratified analyses.

Continuous variables are reported as mean ± SD or median (IQR) and categorical variables as counts (percentages). Group differences used χ^2^ (categorical) and independent-samples *t*-test or one-way ANOVA (continuous), with non-parametric alternatives when assumptions were violated. Correlations of SUM_BBIS used baseline NCCT hematoma volumes. Two-sided *p* < 0.05 was considered significant; effects are given as RR or OR with 95% CIs. Multivariable adjustment for baseline volume, age, IVH, anticoagulation, and management confirmed the independent prognostic value of SUM_BBIS and the individual NCCT signs. All results reflect the retrospective dataset. The study was approved by the Sibiu Emergency Hospital Ethics Committee (Approval No. 91/2025) and conducted in accordance with the Declaration of Helsinki.

## 3. Results

A total of 404 patients with spontaneous intracerebral hemorrhage (ICH) formed the study cohort and underwent baseline and follow-up NCCT scans. The mean composite SUM_BBIS score across the cohort was 1.43. Surgical intervention was performed in 94 (23.3%) patients, with 68 early and 26 delayed. Baseline characteristics by management strategy are detailed in Table 1.

IRS was the most frequent sign (62.1%), followed by SS (34.9%), BHS (31.1%), and BS (15.3%). Mean SUM_BBIS was 1.43, and it was similar between females (1.38) and males (1.46). In exploratory sex-stratified analyses, the prevalence of the four predefined NCCT signs and their associations with hematoma expansion and in-hospital mortality were directionally similar in women and men. Interaction terms (sex×individual sign and sex×SUM_BBIS) were not statistically significant (all *p*-interaction ≥0.05), indicating no evidence of sex-specific modification of these relationships. The Satellite Sign was observed more frequently in our cohort compared with some prior reports, but inter-rater reliability remained substantial (κ >0.70), indicating consistent detection. Mean SUM_BBIS scores increased significantly with hematoma size (small: 0.90; medium: 1.40; large: 2.25; ANOVA *p* < 0.001) and intraventricular hemorrhage presence (IVH: 1.76; see Table 2). Brainstem hemorrhages had the highest mean SUM_BBIS (2.33), while cerebellar hemorrhages had the lowest (0.76). Black Hole Sign varied significantly by location, as it was highest in the brainstem (66.7%) and lowest in cerebellar hemorrhages (14.6%; *p* < 0.001).

**Figure 5:**
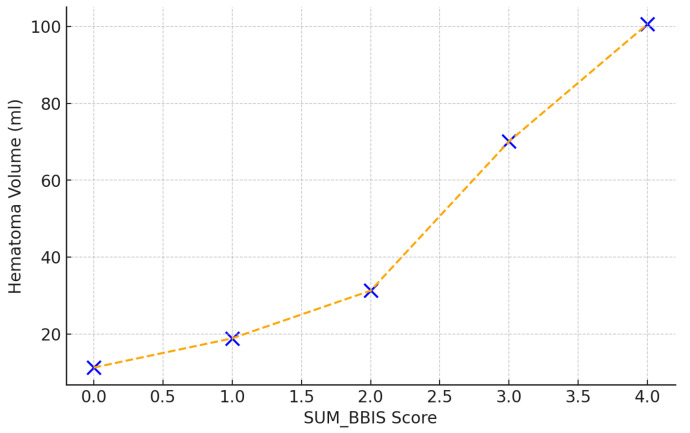
Correlation between SUM_BBIS and baseline hematoma volume (mL); Spearman r = 0.78, *p* < 0.001.

A strong correlation was observed between the composite SUM_BBIS score and baseline hematoma volume (Spearman r = 0.78, *p* < 0.001). Specifically, hematoma volumes increased markedly with each incremental increase in SUM_BBIS, from 11.3 mL at score 0 to 18.9 mL (score 1), 31.3 mL (score 2), 70.1 mL (score 3), and 100.6 mL at the maximum score of 4. This underscores the predictive power of the radiological complexity score regarding hematoma size (Figure 5).

**Figure 6:**
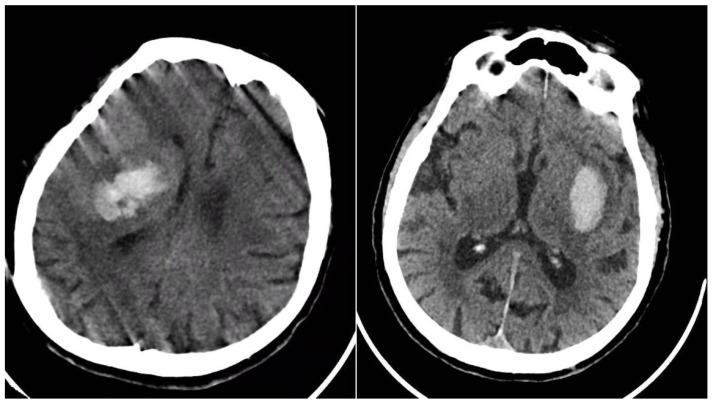
(**Left**) ICH with expansion with the presence of BHS and IRS; (**right**) ICH without hematoma expansion; no NCCT signs observed.

Table 3 presents the hematoma expansion status (YES = 89, NO = 315; total = 404) versus each baseline NCCT sign; column totals exceed 404 because any given patient could display more than one marker. All four signs were markedly over-represented in the expansion group: Blend Sign 28.1% vs. 11.4%, Black Hole Sign 56.2% vs. 23.8%, Irregular Shape 75.3% vs. 54.9%, and Satellite Sign 50.6% vs. 29.8% (all *p* < 0.001). The strongest association was for the Black Hole Sign, underscoring that discrete intra-hemorrhagic hypodensity denotes active or recurrent bleeding and substantially elevates the risk of volume growth (Figure 6).

**Figure 7:**
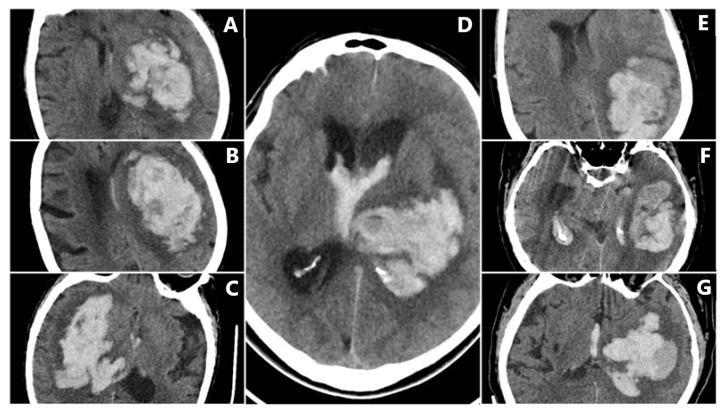
Intracerebral hemorrhage from the subgroup with in-hospital mortality, demonstrating a high incidence of NCCT signs. (**A**–**C**) BHS + IRS + SS. (**D**) BHS + BS + IRS. (**E**–**G**) BS + IRS + SS.

In-hospital mortality analysis demonstrated an even clearer relationship with radiological findings (Figure 7). Patients who died had a mean SUM_BBIS of 1.95, whereas survivors had a mean value of 0.93 (independent-samples *t*-test *p* < 0.001). At the individual sign level, all four markers appeared markedly more often in non-survivors: BS in 24.8% vs. 11.1% of survivors (*p* < 0.001), BHS in 71.0% vs. 15.4% (*p* < 0.001), IRS in 87.2% vs. 50.8% (*p* < 0.001), and SS in 52.0% vs. 27.2% (*p* < 0.001). Table 4 illustrates these proportions. In pairwise comparisons, the Black Hole Sign had the largest absolute gap between fatal and nonfatal cases (50.9 percentage points), suggesting that a large fraction of fatal ICH cases exhibit pronounced intra-hematoma hypodensity indicative of ongoing or repeated bleeding episodes.

**Figure 8:**
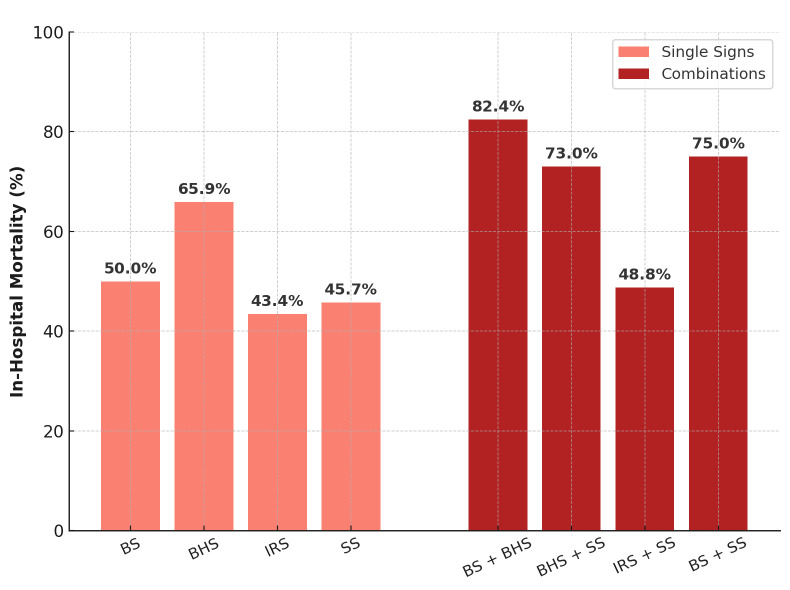
In-hospital mortality (%) by individual NCCT signs and selected sign combinations. Abbreviations: BS = Blend Sign; BHS = Black Hole Sign; IRS = Irregular Shape; SS = Satellite Sign.

Figure 8 and Table 5 illustrates the marked increase in in-hospital mortality when multiple NCCT signs coexist. Mortality notably peaks for combined signs, such as the Blend and Black Hole Sign (82.4%), highlighting their strong cumulative prognostic significance.

**Table 5:** Predictive value of NCCT signs for in-hospital mortality by management strategy.

NCCT Sign	Management	Mortality % (*n/N*)	OR (95% CI)
Blend Sign	Conservative (*n* = 310)	54% (25/46)	3.3 (1.8–6.0)
	Surgical (*n* = 94)	37% (6/16)	1.2 (0.4–3.3)
Black Hole Sign	Conservative (*n* = 310)	71% (67/94)	17.4 (9.4–32.0)
	Surgical (*n* = 94)	50% (16/32)	3.1 (1.3–7.4)
Irregular Shape	Conservative (*n* = 310)	45% (83/184)	3.9 (2.3–6.4)
	Surgical (*n* = 94)	39% (26/67)	1.9 (0.8–4.3)
Satellite Sign	Conservative (*n* = 310)	55% (52/95)	4.3 (2.6–7.0)
	Surgical (*n* = 94)	41% (19/45)	2.1 (0.9–4.8)

Surgical candidates exhibited greater radiological complexity at baseline (mean SUM_BBIS 1.70 ± 1.12 vs. 1.35 ± 1.02 in conservatively treated cases, *p* = 0.003), chiefly driven by higher rates of Irregular Shapes (71.3% vs. 59.3%, *p* = 0.04) and Satellite Signs (47.9% vs. 30.7%, *p* = 0.002), while Blend and Black Hole signs were comparable (17.0% vs. 14.8% and 34.0% vs. 30.3%, *p* > 0.05); however, the SUM_BBIS gap lost significance after adjustment for hematoma volume and clinical covariates, indicating that surgical selection relied more on size and location than NCCT markers. Sub-group analysis (Table 6) showed the highest composite scores in chronic kidney disease (1.60) and thrombocytopenia (1.68), moderate elevation with chronic alcohol use (1.50), and the lowest scores in statin users (0.92). Among anticoagulated patients, warfarin produced more heterogeneous bleeding than DOACs (mean SUM_BBIS 1.48 vs. 1.32; Black Hole Sign 59.1% vs. 37.9%), although the overall score difference was not significant (*p* > 0.05). The pronounced Black Hole Sign prevalence in warfarin and DOAC-related ICH supports its association with deeper, unstable hematomas.

Overall, hemorrhages displaying multiple NCCT signs were larger, favored deep or brainstem locations, extended into the ventricles more often, and carried markedly higher risks of expansion and in-hospital death. The same radiological complexity clustered in patients with coagulopathies, warfarin therapy, or thrombocytopenia and in systemic conditions, such as chronic kidney disease or heavy alcohol use. These robust associations support routine, systematic scoring of NCCT signs to flag high-risk ICH early and prompt timely, aggressive management.

## 4. Discussion

In this 404-patient single-center ICH cohort, we quantified four NCCT signs—Blend, Black Hole, Irregular Shape, Satellite—and combined them as SUM_BBIS (0–4). Irregular Shape was most prevalent (62.1%), followed by Satellite (34.4%), Black Hole (30.9%), and Blend (15.1%). Higher SUM_BBIS correlated with larger hematomas, infratentorial (especially brainstem) location, IVH, and coagulopathic/systemic comorbidities (warfarin use, chronic kidney disease, thrombocytopenia). In terms of outcomes, in-hospital deaths had SUM_BBIS 1.95 vs. 0.93 in survivors, with each sign more frequent among non-survivors [17]. The Black Hole Sign showed the steepest mortality gradient (71% vs. 15% (conservative) and 52% vs. 24% (surgical)), underscoring its prognostic weight.

The CTA Spot Sign predicts expansion [18], but CTA was not systematically obtained here, precluding a direct benchmark. This common real-world constraint (emergency/resource-limited care) highlights that purely NCCT markers, particularly BHS, retain strong prognostic value when CTA is unavailable; future studies with parallel CTA could quantify additive or overlapping value.

NCCT markers were prognostically informative yet similarly distributed across treatment strategies; surgically managed patients showed only modestly higher radiological complexity (SUM_BBIS 1.70 vs. 1.35; *p* = 0.03), a difference that disappeared after adjusting for baseline volume, consistent with triage driven chiefly by clinical status, hematoma size, and location rather than morphology. As in prior work linking heterogeneous density/irregularity to active bleeding, hematoma progression was more common when ≥1 sign was present [19], in line with the concept that such heterogeneity reflects multifocal or ongoing bleeding [20]. Mortality rose stepwise with increasing SUM_BBIS, indicating cumulative instability.

Clinically, flagging NCCT markers at admission, especially multiple signs, offers a CTA-independent triage tool. These patients merit close neurological monitoring, aggressive blood pressure control, and timely hemostatic therapy (e.g., prothrombin complex concentrate for warfarin-associated bleeds, antifibrinolytics when indicated). SUM_BBIS, a simple imaging-only composite, is practical when onset time is uncertain and CTA is not routine. While external validation is needed, its stepwise associations with expansion and mortality suggest utility for early risk stratification and trial enrichment.

Our findings align with the prior literature: Blend Sign prevalence (15.1%) fits the 10–20% range and its reported expansion risk (OR ≈ 3–5) [9,17]; the Black Hole Sign (30.9%) is recognized as specific to expansion and poor prognosis, often with anticoagulation and deep bleeds [10,18]. The Satellite Sign (34.4%) was somewhat higher than the 15–25% reported elsewhere, plausibly reflecting CT acquisition parameters (e.g., slice thickness, window/level standardization) and a predominance of deep hemorrhages; reproducibility was acceptable [21]. Irregular Shape, the most common sign in our cohort (62.1%), may reflect hypertensive bleeds with multiple bleeding points. Results for SS agree with the meta-analysis by Yang et al., 2020, confirming its predictive value and supporting broader SS evaluation for prompt risk stratification [22]. The narrative review by Huang et al., 2023 similarly highlights BS, BHS, and IRS as robust predictors; combined as SUM_BBIS, they support systematic NCCT assessment to improve early management [23].

Emerging radiomics/deep learning approaches show high accuracy for expansion prediction [24]. Although our scoring was manual, consistency across methods underscores clinical relevance. SUM_BBIS can complement AI pipelines as (i) an interpretable input alongside texture metrics, (ii) a weak label for semi-supervised learning when outcome labels are sparse, and (iii) a real-time triage/calibration signal to flag high-instability phenotypes.

Limitations include the retrospective design, the single-center setting, the reliance on in-hospital mortality rather than a 90-day modified Rankin Scale due to incomplete follow-up (a major limitation), potential underestimation of expansion with early surgery, later presentations affecting the visibility of signs (e.g., Blend), subjective assessment of mild irregularities, and incomplete retrieval of key blood pressure data. Nonetheless, BHS consistently predicted hematoma growth, reinforcing its role as a marker of ongoing bleeding.

Regarding generalizability, our Romanian cohort reflects local patterns (high hypertension burden; relatively frequent VKA use compared with newer registries where DOAC-associated ICH is more prominent), while chronic kidney disease and alcohol-related ICH rates appear similar to European averages. These differences underscore the need for external validation of SUM_BBIS in larger, multinational cohorts before universal adoption [18,19,20].

## 5. Conclusions

In this single-center study of 404 patients with spontaneous intracerebral hemorrhage, four non-contrast CT signs (Blend Sign, Black Hole Sign, Irregular Shape, and Satellite Sign) were consistently associated with hematoma expansion and in-hospital mortality. While all signs correlated with worse outcomes, the Black Hole Sign emerged as the most potent individual predictor of hematoma enlargement. Larger bleeds, deeper location, and intraventricular extension were particularly likely to show multiple radiological signs, reflecting greater hemorrhage complexity. Our findings support routine, systematic assessment of these NCCT markers in acute ICH to identify high-risk patients who may benefit from more aggressive monitoring and earlier intervention.

## Figures and Tables

**Table 1:** Baseline characteristics according to initial management strategy.

Variable	Conservative (*n* = 310)	Surgical (*n* = 94)	*p*-Value
Age (mean, years)	69.3	66.1	0.05
Hematoma volume (median, mL)	24.3	55.4	<0.001
IVH present, n (%)	124 (40%)	37 (39%)	0.91
Blend Sign (BS), n (%)	46 (14.8%)	16 (17.0%)	0.56
Black Hole Sign (BHS), n (%)	94 (30.3%)	32 (34.0%)	0.46
Irregular Shape (IRS), n (%)	184 (59.3%)	67 (71.3%)	0.04
Satellite Sign (SS), n (%)	95 (30.7%)	45 (47.9%)	0.002
SUM_BBIS (mean ± SD)	1.35 ± 1.02	1.70 ± 1.12	0.003

**Table 2:** SUM_BBIS scores by hematoma characteristics.

Characteristic	SUM_BBIS (Mean)
Overall (*N* = 404)	1.43
Large hematoma (>30 mL)	2.13
Medium hematoma (15–30 mL)	1.35
Small hematoma (<15 mL)	0.91
Infratentorial (overall)	1.57
Cerebellum	0.76
Brainstem	2.33
Thalamic	1.34
Lobar	1.11
Capsulo-lenticular	1.23
Intraventricular extension (IVH)	1.76
Male vs. female	1.46 vs. 1.38

**Table 3:** Relationship between NCCT signs and hematoma expansion.

Sign	Expansion YES (*n* = 89)	Expansion NO (*n* = 315)	Total (*N* = 404)	*p*-Value
Blend Sign (BS)	25 (28.1%)	36 (11.4%)	61	<0.001
Black Hole Sign (BHS)	50 (56.2%)	75 (23.8%)	125	<0.001
Irregular Shape (IRS)	67 (75.3%)	173 (54.9%)	240	<0.001
Satellite Sign (SS)	45 (50.6%)	94 (29.8%)	139	<0.001

**Table 4:** Radiological signs in survivors vs. in-hospital deaths.

Sign	In-Hospital Deaths (%)	Survivors (%)	*p*-Value (Chi-Square)
Blend Sign (BS)	24.8	11.1	<0.001
Black Hole Sign (BHS)	71.0	15.4	<0.001
Irregular Shape (IRS)	87.2	50.8	<0.001
Satellite Sign (SS)	52.0	27.2	<0.001

**Table 6:** NCCT sign frequencies and SUM_BBIS in selected comorbidities.

Subgroup	BS (%)	BHS (%)	IRS (%)	SS (%)	SUM_BBIS
Anticoagulant therapy (overall)	11.3	47.2	54.7	31.3	1.36
–Vitamin K antagonists (VKA)	9.0	59.1	59.0	28.5	1.48
–DOAC	13.8	37.9	55.2	35.7	1.32
Antiplatelet therapy	15.0	30.0	60.0	30.0	1.22
Statin therapy	0	22.7	59.0	27.2	0.92
Chronic alcohol consumption	16.4	32.7	69.0	41.5	1.50
Chronic kidney disease	12.2	46.9	71.4	43.7	1.60
Diabetes mellitus (type 1 or 2)	8.2	31.1	57.4	30.0	1.22
Thrombocytopenia	11.1	48.1	70.4	44.4	1.68

## Data Availability

De-identified data for this analysis are available at Zenodo (DOI: https://doi.org/10.5281/zenodo.15368709). This cohort is derived from a single-center ICH registry previously characterized for prognostic factors and management outcomes [25,26].

## Abbreviations

The following abbreviations are used in this manuscript:

ICH	Intracerebral hemorrhage
NCCT	Non-contrast computed tomography
BS	Blend Sign
BP	Blood pressure
BHS	Black Hole Sign
IRS	Irregular Shape
SS	Satellite Sign
SUM_BBIS	Sum of Blend Sign, Black Hole Sign, Irregular Shape, and Satellite Sign (range 0–4)
IVH	Intraventricular hemorrhage
DOAC	Direct oral anticoagulant
VKA	Vitamin K antagonist
ANOVA	Analysis of variance
CT	Computed tomography
CTA	CT angiography
ED	Emergency department
